# Frailty as an Independent Risk Factor for Depression in Patients With End-Stage Renal Disease: A Cross-Sectional Study

**DOI:** 10.3389/fmed.2022.799544

**Published:** 2022-02-15

**Authors:** Chun-Yi Chi, Szu-Ying Lee, Chia-Ter Chao, Jenq-Wen Huang

**Affiliations:** ^1^Nephrology Division, Department of Internal Medicine, National Taiwan University Hospital Yunlin Branch, Douliu, Taiwan; ^2^Nephrology Division, Department of Internal Medicine, National Taiwan University Hospital, Taipei, Taiwan; ^3^Graduate Institute of Toxicology, National Taiwan University College of Medicine, Taipei, Taiwan; ^4^Nephrology Division, Department of Internal Medicine, National Taiwan University College of Medicine, Taipei, Taiwan

**Keywords:** chronic kidney disease, depression, end-stage renal disease, frailty, geriatric phenotype, malnutrition, sarcopenia

## Abstract

**Background:**

Depression confers substantial disease burden globally, especially among those with chronic kidney disease (CKD). The presence of depression significantly impairs one's quality of life. Risk factors for depression in patients with CKD remain under-appreciated, and whether frailty, a geriatric phenotype, constitutes a risk factor for depression in this population is unknown.

**Methods:**

We prospectively enrolled patients with end-stage renal disease (ESRD) undergoing hemodialysis for >3 months from National Taiwan University Hospital Yunlin Branch between 2019 and 2021. Clinical, physical, functional, and performance parameters were recorded, followed by frailty/sarcopenia assessment. Depression was screened for using the Geriatric Depression Scale. We analyzed the independent relationship between frailty and depression in these patients, using multiple regression analyses.

**Results:**

Totally 151 patients with ESRD were enrolled (mean 61.1 years, 66.9% male), among whom 16.6% had screening-identified depression. ESRD participants with depression did not differ from those without regarding most parameters except serum creatinine, functional indices, and sarcopenia/frailty status. We found that having greater frail severities was independently associated with a higher probability of depression; having FRAIL- (odds ratio [OR] 5.418) and SOF-based (OR 2.858) frailty independently correlated with a higher depression probability. A linear relation exists between a greater frail severity and the probability of depression. Using a more relaxed criterion for detecting depression, higher SOF scores remained significantly associated with an increased depression risk.

**Conclusions:**

In patients with CKD, frailty independently correlated with a higher probability of having depression. Strategies aiming to attenuate frailty may be able to benefit those with depression simultaneously in this population.

## Introduction

Depression, characterized by an emotional turbulence presenting with somatic, cognitive, and behavioral symptoms, is one of the common psychiatric disorders that affect billions of people and confer substantial disease burden globally (1). Depression is frequently accompanied by loss of interest toward activities and relationships, and prominently impairs an individual's quality of life. Depression exhibits an increased incidence in patients with chronic kidney disease (CKD) and especially those with end-stage renal disease (ESRD), up to 20% to 40% depending upon countries and assessment tools (2). Depression increases CKD patients' long-term mortality by at least 50%, based on National Health and Nutrition Examination Survey (NHANES) results (3). Besides its effect on survival, depression poses a plethora of adverse influences in this population; depressed patients with CKD were found to have a higher incidence of muscle wasting and correlated with a greater degree of functional impairment, according to findings from the Dialysis Outcomes and Practice Patterns Study (DOPPS) (4, 5). Furthermore, depression and its predisposing traits likely modulate the incidence of CKD and its subsequent progression. A recent Mendelian randomization study showed that genetic alleles intimately associated with depressive symptoms conferred a greater risk of carrying lower estimated glomerular filtration rates (eGFRs) (6). Having depressive symptoms places patients at risk of developing accelerated renal function decline, rendering the identification of depression instrumental (7).

Risk factors for depression in patients with CKD or ESRD remain under-appreciated. Existing studies mostly involve patients with depression but without CKD; systematic reviews and meta-analyses indicated that smoking, higher body mass index (BMI), lower blood pressure, personal traits, chronic diseases, and sleep disturbance were associated with an increased risk of having depression in various populations (8, 9). On the other hand, these risk features may not be applicable to those with CKD. Anecdotal studies revealed that severe pain, negative illness perception, and inadequate self-esteem significantly correlated with the presence of depression in those with non-dialysis CKD (10). A longer dialysis vintage also modulated the probability of developing depression in patients with ESRD (11). Since the presence of CKD is associated with premature biological aging (12), emerging studies suggest that geriatric phenotypes demonstrate a high prevalence in this population. Frailty, in particular, is found to be highly prevalent in patients with renal insufficiency. Frailty is recently shown to correlate with the presence and severity of depression in community-dwelling older adults (13), but very few address the possibility whether frailty constitutes a risk factor for depression in patients with CKD. Such relationship has been hypothesized before (14) but never tested in this population. To answer this question, we used a prospectively collected cohort of patients with ESRD to analyze the connection between frailty and depression, using well-validated instruments.

## Subjects, Materials, and Methods

### Ethical Statement

The protocol of the current project has been approved by the institutional review board of National Taiwan University Hospital (No. 201910100RINA). The details of the study protocol adhered to the Declaration of Helsinki, and all participants provided written informed consent.

### Recruitment of Participants and Study Procedures

Patients with ESRD, defined as having an eGFR <15 mL/min/1.73 m^2^, undergoing hemodialysis for more than 3 months, were prospectively enrolled from the dialysis units of National Taiwan University Hospital Yunlin Branch, Douliou and Huwei branches between August 2019 and July 2021. We used the 4-variable Modification of Diet in Renal Disease (MDRD) formula for calculating eGFR. After providing informed consent, participants underwent a 3-step assessment; first, they were interviewed by dedicated nursing staff, with their demographic information (age, gender, and education level) and morbidities recorded. Second, participants underwent physical examination, with their anthropometric parameters [body weight (BW)/body height (BH), waist circumference (WC), and arm/leg circumference] and physical examination indices [blood pressure (BP), heart rate (HR), and respiratory rate] collected (15). At this stage, participants were also instructed to complete performance assessment involving upper and lower limbs, including grip strength (using a TKK dynamometer; Takei Inc., Niigata, Japan), timed chair stand (TCS), time-up-and-go (TUG), and gait speed, according to protocols published previously (16, 17). For all performance assessment, results were obtained after averaging data from 3 repetitive tests. Finally, as the last step, dedicated staff counseled with the participants and administered self-report questionnaires including functional evaluation (eastern cooperative oncology group [ECOG], Karnofsky performance scale, Barthel index, Katz index, and Lawton-Brody instrumental activity of daily living [IADL]), sarcopenia assessment (SARC-F questionnaire), frailty status evaluation [Edmonton frail scale (EFS), Study of Osteoporotic Fractures (SOF) scale, and Fatigue, Resistance, Ambulation, Illness, Loss of weight (FRAIL) scale], and nutritional/appetite screening [Council of Nutrition Assessment Questionnaire (CNAQ)]. The validity of instruments for evaluating frailty, sarcopenia, and nutritional levels in this study has been tested and reassured in patients with CKD and ESRD based on other reports and our prior findings (17–19). Those with a SARC-F score ≥4 were defined as having sarcopenia, while those with a SOF, FRAIL, or EFS score ≥2, 3, and 8 were considered frail, respectively, according to their original schemes. Finally, at least 10 mL of peripheral blood was obtained from participants, sent for laboratory tests including hemogram, serum biochemistry and electrolytes, and metabolic parameters (glucose, lipid profile, and uric acid).

### Outcome Assessment

In this study, we screened these patients regarding whether they had depression, using the Geriatric Depression Scale-15 items (GDS-15). GDS-15 has been recommended as a useful tool to screen for depression in older adults during acute and chronic settings, and also in patients with CKD (20, 21), with a score range between 0 and 15. Compared with other GDS instruments with different item counts (GDS-30, GDS-5, and GDS-4), GDS-15 exhibited a better recognition accuracy compared to others, owing to its advantages of preserving core messages while optimizing the amount of item load (22). GDS-15 assesses participants' depressive symptoms, psychosocial activities, life satisfaction, etc., all of which correlate closely with each other (23). After reassuring patients' cognitive status and literacy level, participants completed the GDS-15 questionnaire, consisting of 10 and 5 positive and negative responses to the presence of depression, respectively, with or without assistance from dedicated staff. Those with a GDS-15 score ≥10 were identified as potentially having depression, according to the existing literature (21–23).

### Statistical Analysis

For continuous variables, we compared between groups using Student's *t*-tests (if normally distributed) or Mann-Whitney U-test (if skewed distribution). For categorical variables, we compared between groups using Chi-square tests. In all analyses, a *p*-value < 0.05 was considered statistically significant. We used IBM SPSS Statistics for Windows, Version 19.0 (Armonk, NY; IBM Corp.) in all statistical analysis.

After completing all assessments in phases 1 and 2, we divided participants into those with and without potential depression, followed by comparing their demographic profiles, comorbidities, physical examination and anthropometric parameters, performance indicators, and laboratory findings. We further examined whether there were differences between those with and without depression, regarding their functional status, frailty, sarcopenia, and nutritional status based on relevant tools. Subsequently, we used multiple regression analysis with having depression or not as the dependent variable with stepwise backward variable selection, incorporating variables with significant differences in univariate analyses. Independent variables were expressed in odds ratios (ORs) with 95% confidence intervals and the associated *p-*values provided. Sensitivity analyses were planned *a priori*, including the adjustment of variable input style (categorical vs. continuous). We also tested whether the replacement of dependent variable, depression or not based on having GDS-15 ≥10, with depression or susceptibility status or not based on having GDS-15 ≥5, might influence our findings.

## Results

During the study period, we enrolled a total of 151 patients with ESRD under chronic hemodialysis, with a mean age of 61.1 years and 66.9% male (Table 1). The most common comorbidity among study participants was hypertension (80.1%), followed by diabetes mellitus (47.0%) and peptic ulcer (30.5%). Nearly half of these patients had chronic pain (43.7%). Participants exhibited on average fair upper and lower limb performance, with a mean TCS, TUG and gait speed of 14.9 s, 10.7 s, and 0.77 m/s, respectively (Table 1). Participants with ESRD were mildly anemic, but had normal electrolyte panels. Their serum uric acid (7.8 mg/dL) and triglyceride (172.2 mg/dL) levels were mildly increased, and participants had mild hyperglycemia (118.7 mg/dL).

**Table 1:** Baseline characteristics of patients with end-stage renal disease enrolled in this study.

	**Total** **(*n* = 151)**	**Without** **depression** **(*n* = 126)**	**With** **depression** **(*n* = 25)**	***p*-value**
**Basic data**				
Age (years)	61.1 ± 12.0	61.0 ± 11.4	61.5 ± 14.7	*0.863*
Sex (male %)	101 (66.9)	86 (68.3)	15 (60.0)	*0.426*
**Education**				*0.284*
None	16 (10.6)	12 (9.5)	4 (16.0)	
Elementary school	32 (21.2)	24 (19.0)	8 (32.0)	
High school	84 (55.6)	74 (58.7)	10 (40.0)	
College or higher	19 (12.6)	16 (12.7)	3 (12.0)	
**Comorbidity**				
Diabetes mellitus (%)	71 (47.0)	58 (46.0)	13 (52.0)	*0.588*
Hypertension (%)	121 (80.1)	102 (81.0)	19 (76.0)	*0.574*
Cirrhosis (%)	6 (4.0)	5 (4.0)	1 (4.0)	*0.994*
Coronary artery disease (%)	32 (21.2)	25 (19.8)	7 (28.0)	*0.365*
Acute myocardial infarction (%)	8 (5.3)	6 (4.8)	2 (8.0)	*0.512*
Heart failure (%)	34 (22.5)	26 (20.6)	8 (32.0)	*0.217*
Peripheral vascular disease (%)	6 (4.0)	4 (3.2)	2 (8.0)	*0.262*
Atrial fibrillation (%)	1 (0.7)	1 (0.8)	0 (0)	*0.658*
COPD (%)	6 (4.0)	5 (4.0)	1 (4.0)	*0.994*
Rheumatology disorders (%)	5 (3.3)	4 (3.2)	1 (4.0)	*0.834*
Malignancy (%)	17 (11.3)	13 (10.3)	4 (16.0)	*0.415*
Peptic ulcer (%)	46 (30.5)	37 (29.4)	9 (36.0)	*0.513*
Prior cerebrovascular accident (%)	9 (6.0)	7 (5.6)	2 (8.0)	*0.640*
Hemiplegia (%)	2 (1.3)	2 (1.6)	0 (0)	*0.529*
Chronic pain (%)	66 (43.7)	52 (41.3)	14 (56.0)	*0.177*
**Physical examination**				
Blood pressure—systolic (mmHg)	147.0 ± 28.9	147.7 ± 29.1	143.6 ± 28.7	*0.516*
Blood pressure—diastolic (mmHg)	71.0 ± 13.6	71.8 ± 14.0	67.0 ± 10.9	*0.108*
Heart rate (per min)	75.8 ± 11.8	75.8 ± 11.6	75.6 ± 13.1	*0.943*
Respiratory rate (per min)	16.8 ± 1.8	16.8 ± 1.8	16.6 ± 1.5	*0.613*
**Anthropometric parameters**				
Body weight (kg)	63.8 ± 15.1	63.1 ± 14.5	67.1 ± 18.0	*0.226*
Body height (cm)	162.9 ± 8.3	162.7 ± 8.2	163.5 ± 8.9	*0.662*
Body mass index (kg/m^2^)	23.9 ± 4.3	23.7 ± 4.2	24.8 ± 5.0	*0.215*
Waist circumference (cm)	86.8 ± 12.9	86.8 ± 13.0	87.1 ± 12.8	*0.906*
Mid-arm circumference (cm)	26.9 ± 4.1	26.9 ± 3.9	26.9 ± 4.9	*0.992*
Mid-leg circumference (cm)	31.9 ± 4.0	31.9 ± 3.8	32.0 ± 5.1	*0.913*
**Performance indicators**				
Grip strength (lb)	155.7 ± 66.6	159.3 ± 63.5	137.5 ± 79.3	*0.136*
Timed chair stand (s)	14.9 ± 9.8	14.8 ± 10.4	15.1 ± 5.3	*0.892*
Timed up and go (s)	10.7 ± 3.4	10.5 ± 3.0	11.4 ± 5.0	*0.243*
Gait speed (m/s)	0.77 ± 0.15	0.77 ± 0.15	0.77 ± 0.13	*0.918*
**Laboratory profile**				
Hemogram				
Leukocyte (K/μL)	7.1 ± 7.6	7.2 ± 8.3	6.5 ± 2.3	*0.689*
Hemoglobin (g/dL)	10.5 ± 1.5	10.5 ± 1.4	10.8 ± 1.9	*0.273*
Platelet (K/μL)	169.5 ± 54.1	167.4 ± 50.6	179.6 ± 69.6	*0.305*
**Biochemistry**				
Urea nitrogen (mg/dL)	85.1 ± 19.8	84.5 ± 20.3	87.8 ± 17.7	*0.445*
Creatinine (mg/dL)	12.3 ± 2.4	12.5 ± 2.2	11.3 ± 2.7	*0.020*
Albumin (mg/dL)	4.0 ± 0.3	4.0 ± 0.3	4.0 ± 0.3	*0.898*
Sodium (meq/L)	136.3 ± 3.0	136.4 ± 2.9	135.6 ± 3.7	*0.202*
Potassium (meq/L)	4.7 ± 0.7	4.7 ± 0.7	4.8 ± 0.7	*0.435*
Calcium (mmol/L)	2.4 ± 0.2	2.4 ± 0.2	2.4 ± 0.2	*0.252*
Phosphate (mg/dL)	5.2 ± 1.6	5.2 ± 1.7	5.1 ± 1.4	*0.854*
**Metabolic**				
Uric acid (mg/dL)	7.8 ± 1.8	7.8 ± 1.8	7.6 ± 2.1	*0.622*
Total cholesterol (mg/dL)	151.9 ± 40.7	151.0 ± 38.6	156.4 ± 50.4	*0.543*
Triglyceride (mg/dL)	172.2 ± 138.0	171.3 ± 143.7	177.2 ± 106.5	*0.845*
Low density lipoprotein cholesterol (mg/dL)	78.8 ± 29.9	78.2 ± 29.0	82.0 ± 34.8	*0.569*
Fasting glucose (mg/dL)	118.7 ± 51.8	116.1 ± 47.5	131.8 ± 69.2	*0.166*

*COPD, chronic obstructive pulmonary disease*.

Among all, 16.6% participants were found to have depression after screening questionnaire use. ESRD participants with potential depression did not differ from those without regarding their demographic profiles, comorbidities, physical examination parameters, anthropometric indices, performance indicators, and most laboratory data except lower serum creatinine levels (*p* = 0.02) among the former group (Table 1).

### Functional Evaluation and Frailty/Sarcopenia Assessment for Study Participants

During functional assessment, participants with ESRD were found to have minor impairment in their activity of daily living, with an average ECOG, Karnofsky performance indicators, and Barthel index scores of 0.97 out of 4, 82.8 out of 100, and 91.3 out of 100, respectively (Table 2). Approximately 17.2% participants had sarcopenia, while 12.6, 19.2, and 23.8% of them had FRAIL-, EFS-, and SOF-defined frailty, respectively. Participants with depression had significantly higher ECOG (*p* = 0.028) and instrumental activity of daily living scores (*p* = 0.034) but lower Karnofsky performance indicators (*p* = 0.006) (Table 2). Those with depression were significantly more likely to have sarcopenia than those without (*p* = 0.032). Similarly, those with depression had a significantly higher prevalence of frailty (40–52%) compared to those without (7.1–18.3%) (Table 2). Participants without depression had better appetite in the form of higher CNAQ scores than those without (*p* = 0.033).

**Table 2:** Functional and geriatric syndrome evaluation results of study participants.

	**Total** **(*n* = 151)**	**Without** **depression** **(*n* = 126)**	**With** **depression** **(*n* = 25)**	***p*-value**
**Functional evaluation**				
ECOG	0.97 ± 0.77	0.91 ± 0.73	1.28 ± 0.89	*0.028*
Karnofsky performance indicators	82.8 ± 14.6	84.2 ± 13.7	75.6 ± 16.9	*0.006*
Barthel index scores	91.3 ± 22.2	92.8 ± 20.8	83.6 ± 27.6	*0.058*
Katz index scores	5.4 ± 1.6	5.5 ± 1.5	4.9 ± 2.1	*0.083*
Lawton-Brody IADL scores	1.7 ± 2.3	1.6 ± 2.2	2.6 ± 2.6	*0.034*
**Sarcopenia**				
SARC-F scores	1.6 ± 2.5	1.4 ± 2.4	2.9 ± 3.0	*0.006*
Sarcopenia	26 (17.2)	18 (14.3)	8 (32.0)	*0.032*
**Frailty**				
Edmonton frail scale scores	5.1 ± 2.6	4.8 ± 2.4	6.8 ± 2.8	*<0.001*
EFS-defined frailty (%)	29 (19.2)	19 (15.1)	10 (40.0)	*0.004*
FRAIL scale scores	0.90 ± 1.26	0.75 ± 1.10	1.68 ± 1.68	*0.001*
FRAIL-defined frailty (%)	19 (12.6)	9 (7.1)	10 (40.0)	*<0.001*
SOF scale scores	1.00 ± 0.76	0.90 ± 0.73	1.56 ± 0.71	*<0.001*
SOF-defined frailty (%)	36 (23.8)	23 (18.3)	13 (52.0)	*<0.001*
**Nutritional evaluation**				
CNAQ scores	26.9 ± 3.1	27.1 ± 2.9	25.7 ± 3.8	*0.033*

*CNAQ, Council on Nutrition Appetite Questionnaire; ECOG, Eastern Cooperative Oncology Group; EFS, Edmonton frail scale; IADL, instrumental activity of daily living; SOF, Study of Osteoporotic Fracture*.

### Independent Predictors of Depression in Patients With End-Stage Renal Disease

We then conducted multiple regression analyses to uncover independent factors associated with having depression after the screening test in study participants. After accounting for variables with significant between-group differences in univariate analysis (Tables 1, 2), including serum creatinine, functional evaluation results (Karnofsky and IADL scores), SARC-F scores, frailty scores (EFS, FRAIL, and SOF scales), and CNAQ scores, we found that having a greater frail severity, including higher EFS (OR 1.365, 95% CI 1.057–1.762) and SOF scores (OR 3.076, 95% CI 1.458–6.493), was independently associated with a higher risk of developing potential depression (Table 3). Sensitivity analyses were done using having frailty or not based on EFS, FRAIL, and SOF scales replacing frailty-assessing scores; we similarly revealed that having FRAIL- (OR 5.418) and SOF-based (OR 2.858) frailty independently correlated with a higher depression probability (Table 3). Alternatively, we used a more relaxed criterion, having a GDS ≥5, as the dependent variable in another set of regression analysis; we discovered that higher SOF scores remained significantly associated with an increased risk (Table 3).

**Table 3:** Independent factors associated with having depression among patients with end-stage renal disease.

**Variables^&^**	**Odds ratio**	**95% confidence interval**	***P*-value**
**Having depression, incorporating frailty scores**			
EFS scores (per 1 score)	1.365	1.057–1.762	*0.017*
SOF scores (per 1 score)	3.076	1.458–6.493	*0.003*
**Having depression, incorporating frailty status**			
FRAIL-based frailty	5.418	1.723–17.032	*0.004*
SOF-based frailty	2.858	1.032–7.914	*0.043*
**Having depression or depression susceptibility**			
SOF scores (per 1 score)	3.517	1.642–7.532	*0.001*

& *Incorporating variables with significant differences in univariate analyses, including serum creatinine, Karnofsky score, SARC-F scores, IADL scores, Edmonton frail scale scores (or frailty status), SOF scores (or frailty status), FRAIL scale scores (or frailty status), and Council of Nutrition Assessment Questionnaire scores*.

Based on our results that the prevalence of potential depression in those without and with SOF-defined frailty was 10.4% and 36.1%, respectively, and that the alpha value was set at 0.05, we could derive a *post-hoc* power of 91.8% for detecting difference of a dichotomous endpoint.

## Discussion

In this study, we prospectively enrolled a group of patients with ESRD and comprehensively assessed their baseline clinical, physical, functional, and performance status, followed by depression screening. After adjusting for potential confounders, we were able to show that frailty was an independent factor associated with having depression in these patients, in a graded fashion. This phenomenon serves to remind physicians that frailty evaluation may partially assist them in determining the probability of depression among patients with CKD, and that frailty-curbing strategy may potentially benefit CKD patients with depression as well.

The prevalence of depression ranged between 16.6 and 18.5% in the current study. Compared to results reported by others (25 to 35%) (2), the prevalence of depression was modestly lower; several reasons might be responsible for this phenomenon. First of all, the nutritional status of our study participants appeared fair, with relatively good muscle power and functional status. This assertion is supported by their average BMI (23.9 ± 4.3 kg/m^2^) and fair gait speed/grip strength (Table 1) relative to the mean values obtained previously in Taiwanese patients with ESRD (16). On the other hand, the sensitivity of our depression screening instrument may need to be optimized. There are other ways of detecting depression in patients with CKD, including Beck depression inventory, Hamilton rating scale, major depression inventory, center for epidemiological studies depression screening index, etc. (2), but heterogeneity in results is not uncommon. Specifically, it is speculated that the estimation of depression prevalence may be lower when patients are assessed by clinical interview compared to data obtained by self-report (2). Since our participants were assessed by a hybrid of self-report and clinical interview, it is likely that the prevalence estimate could be somewhat lower. Nonetheless, the relationship between depression identified by different instruments and adverse outcomes remains consistent across tools.

The paths connecting frailty to the inception of depression, though frequently under-recognized, can be complex. Frailty has been proposed to be conductive to having a mindset of suboptimal health perception and inadequate competence in self-care (24); possessing illness perceptions including a greater symptomatology, less personal control, and maladaptive coping strategies has been shown to increase the distress level of patients with CKD (25), predisposing them to the subsequent development of depression. Patients with frailty frequently report the co-presence of other geriatric syndromes such as malnutrition, polypharmacy, and functional impairment. Geriatric phenotypes, including malnutrition and polypharmacy, has been suggested to independently correlate with reporting depressive symptoms (26), serving as another rationale for linking frailty to depression. Alternatively, frailty may co-exist with a greater severity of occult inflammation; a meta-analysis showed that frail patients had significantly higher circulating levels of C-reactive protein and interleukin-6 than non-frail ones (27). Chronic inflammation, or the ingestion of a pro-inflammatory diet, potentially increases the risk of depression (28), constituting another link between frailty and depression. The strength of such link may become more prominent in patients with CKD, whose severity of inflammation outnumbers those without (29). From these arguments, we can presume that frailty may increase the risk of depression in patients with CKD, through multiple mechanisms.

We showed that results generated from one of the three frailty-assessing instruments (SOF scale) exhibited a consistent association with the risk of depression across different models, while the other two (EFS and FRAIL scale) were conditionally associated (Table 3). There are differences regarding the scale components, the predictive accuracy, the ease of administration, and the applicability between the 3 instruments. SOF scale has fewer items, is easier for use, and has been widely validated in various populations for outcome prediction, but it tends to over-screen frailty (30). With these features in mind, it is expectable that SOF may potentially be more sensitive for identifying those with earlier presentations of frailty compared to other instruments; indeed, we found that SOF identified a significantly higher proportion of patients with frailty in our cohort. Prior studies revealed that in certain population, SOF scale exhibited better detection ability for adverse outcomes compared to Cardiovascular Health Study (CHS) scale (31). Therefore, our findings may be reasonable in light of the inherent differences between frailty-detecting instruments.

Existing interventions to ameliorate depressive symptoms in patients with CKD include wellbeing enhancement through counseling or electronic apps (32), exercise regimens such as cycling, strengthening, pilates, jogging or home-based ones (33), mind-body interventions such as yoga or relaxation therapies (34), pharmaceutical options (specific serotonin-selective reuptake inhibitor) (35). However, available options more or less have their disadvantages; patients with CKD already have high pill burden and multimorbidity, which renders them reluctant to receive pharmacological treatments or predisposes them to side effects. Psychiatric services, a quintessential part of depression management, may be unavailable due to staff shortage or uneven distributions, especially for those who do not live in urban areas. It would benefit patients with CKD if more treatment options can be tested for the management of depression in this population. Based on our findings, we propose that frailty-targeted interventions may an alternative choice if we aim to reduce the probability of depression in these patients. For example, dedicated exercise training, comorbidity management, senolytics, etc. may all be potential options for anti-frailty purpose (36). It would be tempting to pursue these options as adjunct options for counteracting depression in patients with CKD, although more studies are needed in this regard.

Our study has its strengths and limitations. In our study, we collected a comprehensive set of variables, ranging from demographic, morbidities, anthropometric, physical, functional, and laboratory parameters, as well as frailty, nutrition, and sarcopenia assessment results. This approach likely reduced the probability of result influenced by most residual confounding factors. We used multiple frailty-assessing instruments to evaluate frailty, and the results were robust. However, limitations do exist. Our sample size was not large, and statistical efficiency might not be sufficient. In addition, as discussed above, the sensitivity of our depression-assessing instrument might vary according to the methods of administration and possibly patient features. For confirming the diagnosis of depression, a psychiatrist evaluation and criteria fulfillment would be needed, but such service could be time-consuming and not readily available anytime. There are opinions suggesting that the utilization of depression screening tests may assist in earlier detection and potentially outcome improvement (37). Therefore, we used the widely applicable GDS to screen for depression in our patients. Our study is cross-sectional in nature, so a causal relationship cannot be ascertained between frailty and depression in these patients. There are theories and investigations showing that depression may also increase the risk of frailty (38), suggesting that a bi-directional relationship potentially exists between frailty and depression. Nonetheless, we could not derive such conclusion based on our results. Finally, our patients with ESRD were of Asian ethnicity and received chronic hemodialysis only, like in our prior experimental and clinical work (39, 40), and extrapolation of our findings to those of other ethnicities or under chronic peritoneal dialysis would not be feasible. Broader inclusion criteria and the involvement of a cohort follow-up design would better answer these questions.

## Conclusion

We prospectively included a cohort of patients with ESRD under chronic hemodialysis and documented their baseline status of depression, using a validated instrument, along with an extensive array of interfering variables. After adjustment, we discovered that the presence of frailty was independently associated with a higher risk of exhibiting depression, while a greater frail severity correlated with an increased risk as well. Although a definitive conclusion cannot be obtained based on the current findings, we believe that the link between frailty and depression truly exists among patients with ESRD, and that strategies aiming to attenuate frailty may be able to benefit those with depression simultaneously.

## Ethics Statement

The studies involving human participants were reviewed and approved by the Institutional Review Board of the National Taiwan University Hospital (No. 201910100RINA). The patients/participants provided their written informed consent to participate in this study.

## Author Contributions

C-TC and J-WH: study design. S-YL, C-TC, and J-WH: data analysis. C-YC, SY-L, C-TC, and J-WH: article drafting. All authors approved the final version of the manuscript.

## Funding

The study was financially sponsored by National Taiwan University Hospital Yunlin Branch (NTUHYL 110.N004) and Ministry of Science and Technology, Taiwan (MOST 109-2314-B-002-193-MY3 and MOST 108-2314-B-002-062-MY3). The sponsors have no role in the study design, data collection, analysis, and result interpretation of this study.

## Conflict of Interest

The authors declare that the research was conducted in the absence of any commercial or financial relationships that could be construed as a potential conflict ofinterest.

## Publisher's Note

All claims expressed in this article are solely those of the authors and do not necessarily represent those of their affiliated organizations, or those of the publisher, the editors and the reviewers. Any product that may be evaluated in this article, or claim that may be made by its manufacturer, is not guaranteed or endorsed by the publisher.

## Abbreviations

BH: body height
BMI: body mass index
BP: blood pressure
BW: body weight
CHS: cardiovascular health study
CI: confidence interval
CKD: chronic kidney disease
CNAQ: Council of Nutrition Assessment Questionnaire
DOPPS: Dialysis Outcomes and Practice Patterns Study
ECOG: eastern cooperative oncology group
EFS: Edmonton frail scale
eGFR: estimated glomerular filtration rate
ESRD: end-stage renal disease
FRAIL, fatigue, resistance, ambulation, illness: and loss of weight
GDS-15: Geriatric Depression Scale-15 items
IADL: instrumental activity of daily living
MDRD: Modification of Diet in Renal Disease
NHANES: National Health and Nutrition Examination Survey
OR: odds ratio
SOF: study of osteoporotic fracture
TCS: timed chair stand
TUG: time up and go
WC: waist circumference.
